# Vaccination with synthetic long peptide and CpG 2395 in AddaVax induces potent anti-tumor effects

**DOI:** 10.3389/ebm.2025.10509

**Published:** 2025-05-06

**Authors:** Shanshan Jiang, Shuqi Zhao, Qiaojiajie Zhao, Yinfang Wang, Weihua Zhang, Yangmeng Feng, Lijie Zhang

**Affiliations:** ^1^ Institute of Hematological Research, Shaanxi Provincial People’s Hospital, Xi’an, China; ^2^ Department of Hematology, Shaanxi Provincial People’s Hospital, Xi’an, China; ^3^ Central laboratory, Shaanxi Provincial People’s Hospital, Xi’an, China

**Keywords:** AddaVax, synthetic long peptides, vaccine, anti-tumor effects, CpG 2395

## Abstract

Cancer/testis antigen HCA587, also known as MAGE-C2, highly expressed in a wide range of malignant tumors with unique immunological characteristics, serves as a potential target for tumor immunotherapy. Synthetic long peptides from HCA587 (HCA587 SLP) were proved to be highly immunogenic and promising in the application of cancer vaccine composed of Freud’s adjuvant (FA) and CpG 1826, whereas, scarce CD8^+^ T cell response may limit their anti-tumor effects during previous research. In this study, notwithstanding the multiple potential of IFN-α in immune modulation, there was no evidence of IFN-α in enhancing the immune response elicited by HCA587 SLP vaccine (HCA587 SLP + FA + CpG 1826). Given the unpleasant side-effects of Freud’s adjuvant, then we applied AddaVax as a substitute for Freud’s adjuvant, and we demonstrated that HCA587 SLP, formulated with AddaVax and CpG 2395, could induce more robust immune response in comparison with combined use of AddaVax and CpG 1826 through ELISpot assay. Furthermore, both IFN-γ-secreting CD4^+^ T cell and CD8^+^ T cell responses could be elicited by HCA587 SLP in combination with AddaVax and CpG 2395, and CD8^+^ T cell response was most obviously observed under the condition of 10-h inhibition of cytokine secretion by brefeldin A post 10-h stimulation with HCA587 SLP, suggesting that cross presentation of exogenous long peptides to CD8^+^ T cells may require more time than direct presentation to CD4^+^ T cells. This vaccine formulation also conferred protection against challenge with HCA587-expressing B16 melanoma presented by delayed tumor growth and prolonged survival compared. This formulation of HCA587 SLP vaccine holds promise for the treatment of patients with cancer in future clinical trials.

## Impact statement

Synthetic long peptide (SLP) vaccines have shown great potential in cancer treatment. However, lack of CD8+ T cell responses restricted their anti-tumor effects. It is well known that CD8+ T cells are the major “killer” in anti-tumor immunity. Therefore, efficient CD8+ T cell responses are the key to maximize the antitumor potential of SLP vaccine. In this study, we applied AddaVax and CpG 2395 as adjuvants to enhance the SLP-specific CD8+ T cell responses and improve the anti-tumor effects. This vaccine formula also appeared to be safer than Freud’s adjuvants by avoiding the unpleasant side-effects such as tubercle and fester. Our research demonstrated the emulsion of SLP and CpG 2395 in AddaVax as a more effective and safer vaccine combination to combat the tumors.

## Introduction

Cancer vaccines aim to stimulate the patient’s adaptive immune system against specific tumor antigens in order to make tumors under control, and induce the long-term memory to prevent the tumor relapse [1–3]. The priority of successful cancer vaccines is to pinpoint the optimal target tumor antigen. Cancer/testis antigen HCA587, also known as MAGE-C2, is widely expressed in cancers including melanoma, hepatocellular carcinoma, bladder cancer, lung cancer, sarcoma, etc., but not normal tissues except testis [4, 5]. HCA587 protein vaccine has been proved to induce humoral and cellular immune responses efficiently, and confer protection in prophylactic and therapeutic animal models during our previous study [6]. All these findings make HCA587 an excellent target antigen for cancer immunotherapy.

Synthetic long peptides (SLP) contain multiple epitopes for presentation on MHC Ⅰ and MHC Ⅱ capable of inducing both CD4^+^ and CD8^+^ T cell responses. SLP also allows for administration to patients independent of their HLA types and avoiding immune tolerance through antigen presentation on non-professional antigen presenting cells (APCs) [7]. SLP is better processed by dendritic cells (DC) than protein, thus leads to enhanced CD8^+^ T cell responses [8]. Moreover, usage of SLP can induce T-cell responses to subdominant epitopes of tumor antigen, thus exerting more broad biological activity against intact tumor cells. All these above mentioned properties endow SLP with an edge in the vaccine field.

Given the advantages of the synthetic long peptide as cancer vaccine, we tested the capacity of HCA587 SLP to stimulate HCA587-specific immune responses and antitumor effects in animal tumor model. Our previous results indicated that HCA587 SLP combined with CFA and CpG 1826 could elicit Th1-type immune response and decrease the tumor incidence, but no protection against developed HCA587-expressing tumors was observed compared to the adjuvant group [9]. The possible reason may lie in the dominating Th1 immune response barely composed of CD8^+^ T cells producing IFN-γ.

As far as we know, CD8^+^ T cells are the major “killer” in anti-tumor immunity, therefore, in order to maximize the antitumor potential of SLP vaccine-induced CD8^+^ T cell response, the optimal adjuvants are in the need for vaccine development.

Many researches had suggested that IFN-α could enhance the cross-priming activity of DC through up-regulation of MHC class Ⅰ and co-stimulatory molecules, thereby eliciting more robust CD8^+^ T cell response [10–12]. The multiple functions of IFN-α, including both direct and indirect anti-tumor effects, have made it suitable for the treatment of a number of cancers [13–15].

Compared to the paraffin oil used in Freund’s adjuvants, Squalene is a kind of oil more readily metabolized. AddaVax is a squalene-based oil-in-water nano-emulsion, which can elicit both cellular (Th1) and humoral (Th2) immune responses, showing as a great potential adjuvant. Except for the depot effect and enhancement of antigen persistence at the injection site, this class of adjuvants can act through recruitment and activation of antigen presenting cells, and direct stimulation of cytokine and chemokine production by macrophages and granulocytes [16–18]. It was reported that peptide vaccines assembled with AddaVax and IFN-α-inducer could bring protection against tumor [19, 20].

Many reports from our and other research centers demonstrated that CpG has proved strong immune-stimulatory effects. It can be classified into three classes, CpG-A, CpG-B, and CpG-C based on their distinct chemical and biophysical properties. CpG-A was shown to be potent inducer of IFN-α production from pDCs, while CpG-B is a strong B cell stimulator. CpG-C (such as CpG 2395 or ODN 2395), which combine the effects of CpG-A and CpG-B, are potent inducers of IFN-α from pDC and strong B cell activators, proving to be more powerful adjuvant in cancer vaccine development [21–24].

In this study, we immunized the C57BL/6 mice with the emulsion of HCA587 SLP and CpG 2395 (source of endogenous IFN-α) with AddaVax to detect the vaccine-induced immune response and applied this emulsion to tumor-bearing mice to investigate anti-tumor effects in the therapeutic animal model.

## Materials and methods

### Mice and cell lines

6 to 8-week-old C57BL/6 mice were obtained from the Laboratory Animal Center of Xi’an jiao tong University. Mice were housed under standard pathogen-free conditions. All animal studies were approved by the Animal Care and Use Committees of Xi’an jiao tong University Health Science Center. Tumor cell line B16-HCA587 was generated by transfection of melanoma B16 cells of C57BL/6 origin by full-length HCA587 cDNA sequence as described previously. The tumor cells were maintained in DMEM with 10% heat-inactivated fetal bovine serum (Invitrogen).

### Antibodies and cytokines

Fluorescence labeled antibodies specific for CD4 and CD8, IFN-γ and Granzyme B were obtained from Biolegend. In all experiments, control mAbs of the respective IgG isotypes were included. rm-IFNα was purchased from pbl assay science.

### Synthetic long peptides (SLP) and adjuvants

The peptides (13 peptides of 25–35 amino acids long with an overlapping of 14 amino acids) representing the HCA587 protein from amino acids 136 to 373, which harbors most of the published MHC class and epitopes were synthesized at the Ontores Biotechnologies Co. (HangZhou). All peptides were >90% pure as indicated by reverse High-performance Liquid Chromatography. Flu peptide STSADQQSLYQNAD (209-212) was used as a control peptide.

Squalene-based oil-in-water adjuvant AddaVax and ODN 2395 VacciGrade™ (5′-tcg​tcg​ttt​tcg​gcg​c:gcg​ccg-3′) were purchased from Invivogen. Complete Freund’s Adjuvant (CFA) and Incomplete Freund’s Adjuvant (IFA) were purchased from Sigma-Aldrich. All-phosphorothioate modified CpG oligonucleotide (CpG ODN) 1826 (5′ –tcc​atg​acg​ttc​ctg​acg​tt-3′) was synthesized by the Shanghai Sangon Biological Engineering & Technology and Service.

### Immunization procedures

C57BL/6 mice were immunized subcutaneously (s.c.) with 100 μL mixture of SLP (20 μg each peptide), 20 μg ODN 2395 VacciGrade™(CpG 2395) or CpG 1826 and AddaVax; or with synthetic long peptide pool (20 μg each peptide) at the volume of 100 μL admixed with 100 μL CFA/IFA in the presence of 20 μg CpG 1826 (subcutaneous injection next to the immunization site), rmIFN-α was administered s. c. twice a week at the dose of 2500 IU each mouse. Each animal received two immunizations at a 3-week interval. For the vaccination procedures involving Freund’s Adjuvants (FA), IFA was used for the second immunization. Controls were set up by immunizing age-matched mice with adjuvants alone. 8 days after second immunization, the mice were euthanized and the splenocytes were harvested for the following experiments.

### IFN-γ ELISPOT assay

After lysing RBCs, splenocytes were resuspended and seeded at a density of 5 × 10^5^ cells per well in a 96-well nitrocellulose plates (MAHA N4550; Millipore) coated with anti-IFN-γ capture Abs (Mabtech), in the presence of HCA587-derived SLP (1.25 μg/mL each peptide). Flu peptide was used as a negative control for HCA587 SLP. After incubation for 20 h at 37°C, cells were removed, and the plates were washed 5 times with PBS, then incubated with a biotinylated anti-mouse IFN-γ detecting Ab (1:1000, 100 μL/well) for 2 h and streptavidin-alkaline phosphatase (1:1000, 100 μL/well) for 1 h at room temperature. After incubation, the dark violet spots were developed by the addition of substrate solution (BCIP/NBT-plus) (100μL/well). The spots displayed on the plate membranes were automatically counted with the ELISPOT reader.

### Intracellular cytokine assay and flow cytometry

Splenocytes were resuspended and seeded at a density of 5 × 10^6^ cells per well in a 24-well plates, in the presence of HCA587-derived SLP (1.25 μg/mL each peptide). For blocking the intracellular cytokine secretion, brefeldin A (5 μg/mL; Biolegend) was added to the cell culture after 3-h or 10-h stimulation with HCA587 SLP, and further cultured for 10 h. Simultaneous addition of brefeldin A and HCA587 SLP to the cell culture was also tested.

The cells were first stained with fluorescence labeled anti-mouse CD4 and CD8 antibodies, then fixed and permeabilized (Biolegend) according to the manufacturer’s instructions, followed by staining with fluorescence-labeled antibodies against IFN-γ, Granzyme B, or isotype-matched control antibodies. Data were collected using a Beckman Coulter cytometer and analyzed using Kaluza software 2.1.

### Evaluation of the anti-tumor effects of HCA587 SLP adjuvanted with CpG 2395 and AddaVax *in vivo*

The effect of HCA587 SLP vaccination (mixture of HCA587 SLP and CpG 2395, AddaVax) on tumor growth and survival was evaluated in therapeutic mode. Groups of C57BL/6 mice (n = 8–11) were inoculated with 2 × 10^4^ B16-HCA587 tumor cells, the day after tumor challenge, they received the HCA587 SLP vaccination treatment (HCA587 SLP + AddaVax + CpG 2395) or just adjuvant control treatment (AddaVax + CpG 2395) according to the immunization procedures as above mentioned. Mouse survival was monitored daily, and the tumor length and width were measured every 2–3 days with a caliper, and calculated using the following formula: (length × width^2^)/2.

### Statistical analyses

All analysis and graphics were done using GraphPad Prism, version 5 for PC (GraphPad Software, San Diego, CA). The statistical significance of differential findings was determined using Student’s *t*-test. Differences in the survival of mice were analyzed using the Kaplan-Meier method, and groups were compared using the log-rank test. Statistical significance was based on a value of *P* < 0.05.

## Results

### The local administration of rmIFN-α could not further improve the immune response elicited by the HCA587 SLP adjuvanted with FA and CpG1826

**FIGURE 1:**
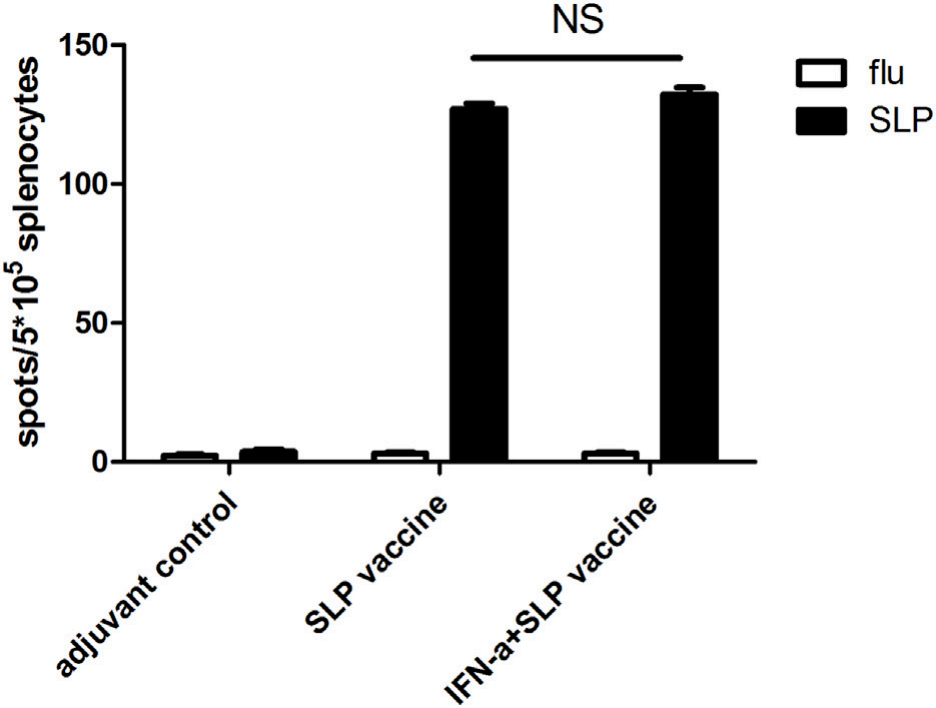
IFN-α could barely improve the immune response induced by SLP vaccine (HCA587 + FA + CpG1826) further. 6-to-8-week female C57BL/6 mice were vaccinated with SLP vaccine (HCA587 + FA + CpG1826) according to a prime-boost procedure (day 0 and day 21), for “INF-α + SLP vaccine” group, recombinant IFN-α (rmIFN-α) was given subcutaneously (s.c.) twice a week at the dose of 2500 IU each mouse. 8 days after the boost vaccination, mice were sacrificed and splenocytes were re-stimulated with HCA587 SLP (1.25 μg/mL each peptide) or irrelevant flu peptide (10 μg/mL) for 20 h. IFN-γ-producing cells were detected by ELISPOT assay. Data were presented as mean ± SD. NS, *P* > 0.05.

Our previous study found that HCA587 SLP vaccine (HCA587+FA + CpG 1826) elicited Th1 immune response, which presented limited effects in reducing the tumor growth and prolonging the survival of tumor-bearing mice. IFN-α has been proved to possess pleiotropic properties, including inhibition of proliferation and angiogenesis and induction of apoptosis [13]. Besides these, IFN-α also exerts immunomodulatory effects such as up-regulation of MHC class I, enhancement of maturation and activation of dendritic cells, thus cross-priming CD8^+^ T cell response, which make it an appropriate candidate to combine with cancer vaccines [11, 12]. To verify whether IFN-α could enhance the immune response elicited by the HCA587 SLP vaccine, we immunized C57BL/6 mice with the recombinant HCA587 SLP formulated with FA and CpG1826, and IFN-α was delivered at the vaccine site twice per week for 4 weeks. Ten days after the second immunization, splenocytes were prepared and analyzed for the presence of HCA587 SLP-specific, IFN-γ-secreting cells using an ELISPOT assay. As depicted in Figure 1, addition of rmIFN-α could barely enhance the HCA587 SLP specific immune response intensity induced by the HCA587 SLP vaccine.

### HCA587 SLP in combination with AddaVax and CpG 2395 (ODN 2395) induce the strong cellular immune response

**FIGURE 2:**
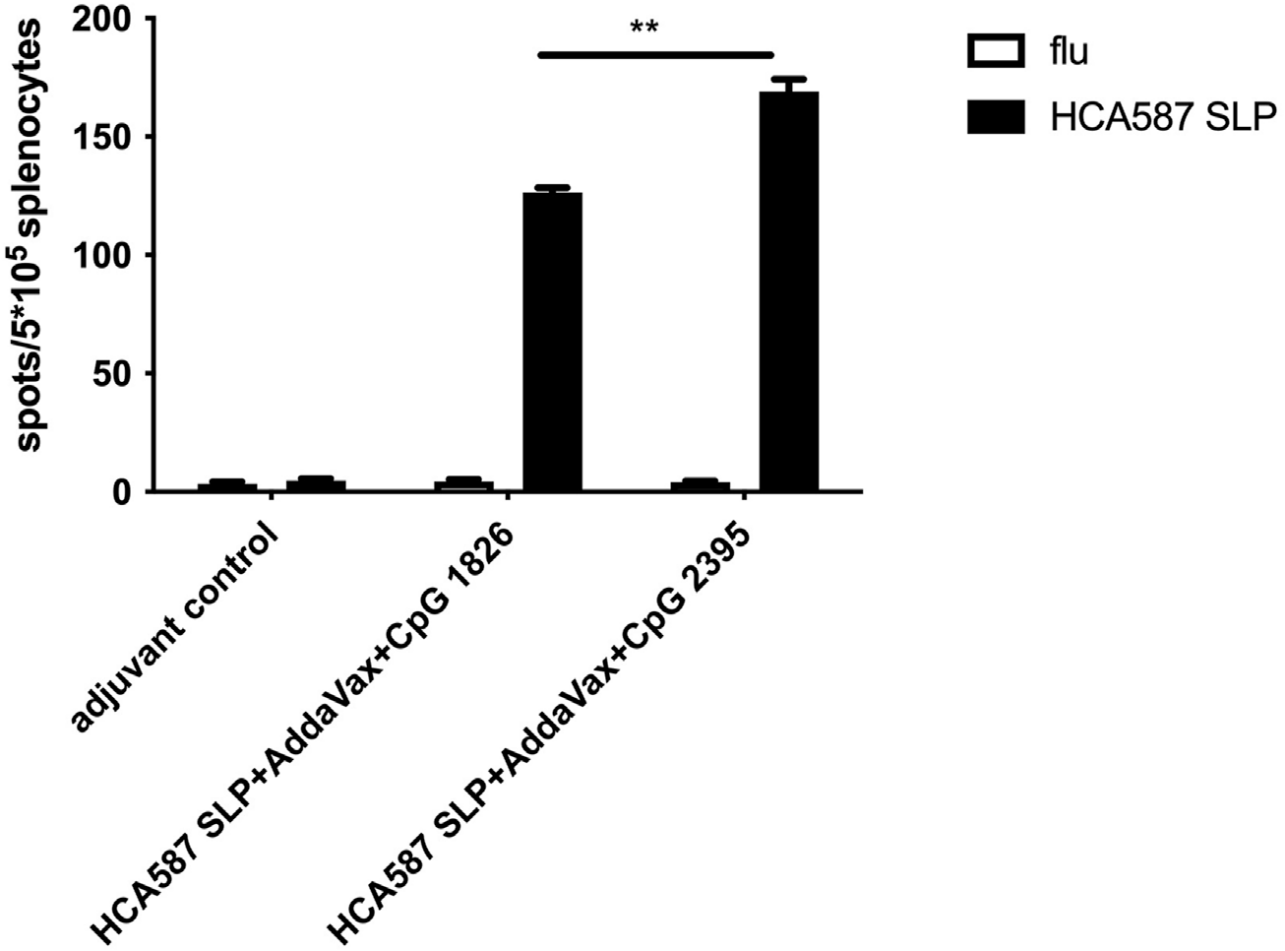
Immunization with HCA587 SLP in combination with AddaVax and CpG induces antigen-specific cellular immune responses. 6-to-8-week female C57BL/6 mice were immunized with HCA587 SLP emulsified in AddaVax with CpG 2395 (20 μg) or CPG 1826 (20 μg) at the base of tail twice 3 weeks away. 8 days after the boost vaccination, mice were sacrificed and splenocytes were re-stimulated with HCA587 SLP (1.25 μg/mL each peptide) or irrelevant flu peptide (10 μg/mL) for 20 h. IFN-γ-producing cells were detected by ELISPOT assay. Data were presented as mean ± SD. **, *P* < 0.01, HCA587 SLP + AddaVax + CpG 2395 in comparison with HCA587 SLP + AddaVax + CpG 1826.

Apart from the inefficiency of rmIFN-α addition in improving HCA587 SLP vaccine invFA + CpG 1826)-elicited immune responses, skin ulcers of vaccinated mice in HCA587 SLP vaccine were frequently observed. This unexpected side effect may be caused by the combined usage of CFA and rmIFN-α, which induced excessive inflammation. AddaVax, a squalene-based oil-in-water nano-emulsion, is more readily metabolized compared to the paraffin oil used in Freund’s adjuvants. AddaVax can elicit both cellular (Th1) and humoral (Th2) immune responses through the activation of the innate immune system such as recruitment and activation of antigen presenting cells, cytokine and chemokine production of innate immune cells. All of these advantages indicated AddaVax as a great potential adjuvant. So we vaccinated C57BL/6 mice with HCA587 SLP emulsified with AddaVax in combination with different types of CpG (CpG 1826 and CpG 2395), thus testified the immune response through ELISPOT assay. As shown in Figure 2, specific cellular immune responses were induced by both types of CpG upon co-application of AddaVax with HCA587 SLP, moreover, a much enhanced cellular response was observed in CpG 2395 group than CpG 1826 group. Thereby the following experiments would adopt the combination of HCA587 SLP with CpG 2395 and AddaVax as the vaccine in the therapeutic model.

### HCA587 SLP adjuvanted with AddaVax and CpG 2395 could induce both CD4^+^ and CD8^+^ T cell responses

**FIGURE 3:**
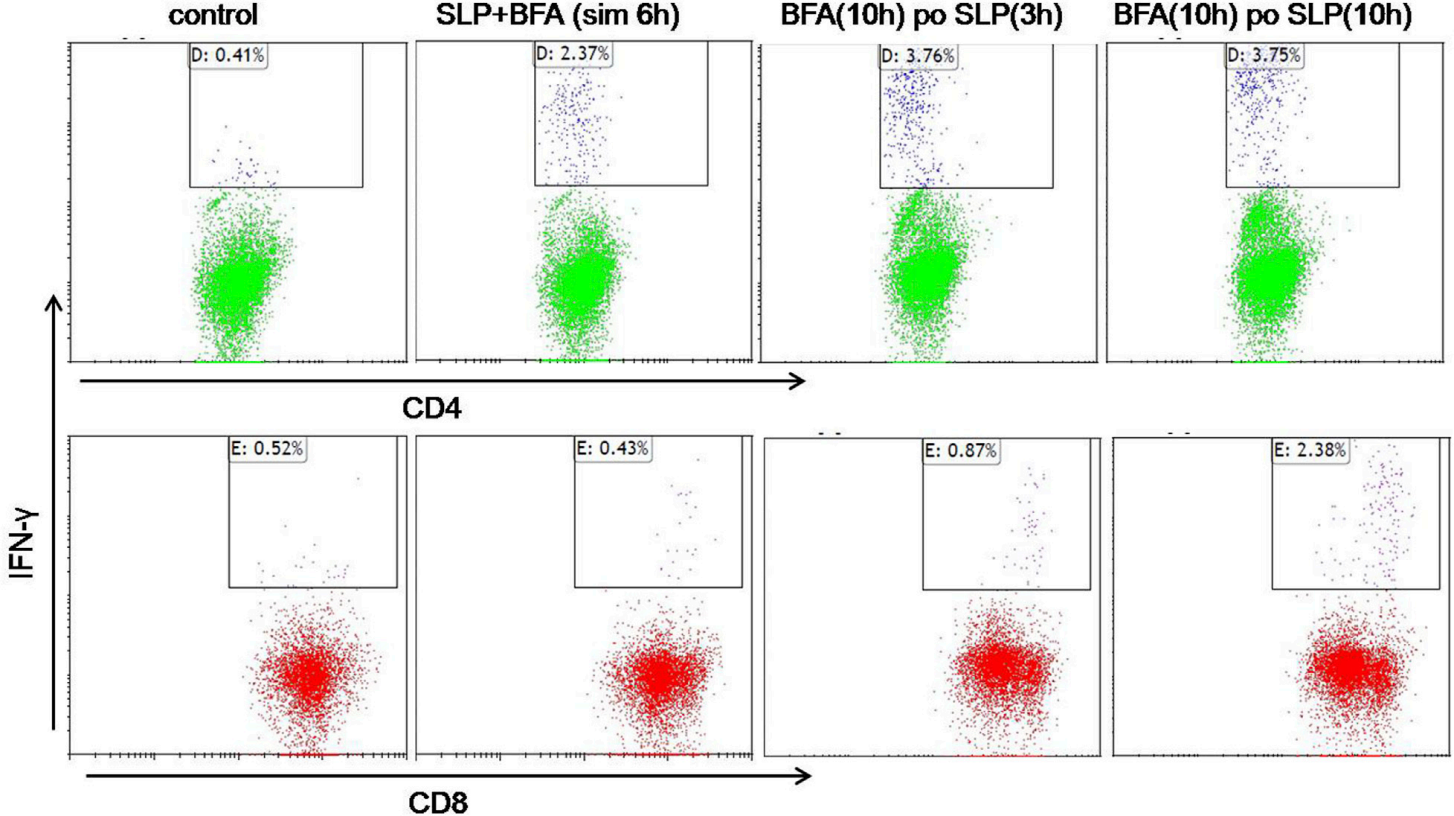
HCA587-specific CD4^+^ T cells and CD8^+^ T cells are induced by HCA587 SLP in combination with AddaVax and CpG 2395. 6-to-8-week female C57BL/6 mice were immunized with the emulsified combination of HCA587 SLP and CpG 2395 with AddaVax at the base of tail twice 3 weeks away. 8 days after the boost vaccination, the splenocytes of mice were stimulated *ex vivo* with HCA587 SLP (1.25 μg/ml each peptide) for ICCS. “BFA(10 h) po SLP(3 h)”, brefeldin A (5 μg/mL) was added to the cell culture after 3-h stimulation with HCA587 SLP, and further cultured for 10 h. “BFA(10 h) po SLP(10 h),” brefeldin A (5 μg/mL) was added to the cell culture post 10-h stimulation with HCA587 SLP, and further cultured for 10 h. “SLP + BFA (sim 6 h),” simultaneous application of brefeldin A and HCA587 SLP was exerted to the splenocytes. The upper right quadrant displayed the positive cytokine-secreting cell population. IgG isotype staining served as control in flow cytometry.

To further verify the efficient production of CD4^+^ T-cell and CD8^+^ T cell responses by HCA587 SLP in combination with AddaVax and CpG 2395, splenocytes from SLP vaccinated mice were re-stimulated with HCA587 SLP *ex vivo* under different conditions, such as simultaneous incubation of SLP and BFA for 6 h, addition of BFA to the cultured splenocytes post 3-h or 10-h stimulation with HCA587 SLP, and 10 h after BFA administration, all the splenocytes were analyzed for the T-cell markers CD4 and CD8 and IFN-γ secretion by multiparameter flow cytometry. As depicted in Figure 3, specific CD4^+^ T-cell responses could be induced independent of the time points of BFA addition, albeit the IFN-γ secreting CD4^+^ T-cells were less upon the simultaneous BFA addition with HCA587 SLP stimulation. However, CD8^+^ T cell responses were most robust when the splenocytes were incubated with HCA587 SLP for 10 h, and then blockade of IFN-γ secretion by BFA for 10 h further. The cause for this discrepancy may lie in that SLP has to be ingested by DC firstly, trimmed to fit into the MHC Ⅰ, and then cross-presented to and primed CD8^+^ T cells, whereas SLP could interact with MHC Ⅱ directly in the endosome, thus displayed at cell surface to activate CD4^+^ T-cells.

### HCA587 SLP combined with AddaVax and CpG 2395 generates antitumor effect in mice challenged with HCA587-expressing tumor cells

The therapeutic tumor model was performed to determine the whether the CD4^+^ and CD8^+^ T cell responses induced by HCA587 SLP immunization with AddaVax and CpG 2395 could translate into anti-tumor effect *in vivo*. B16-HCA587 cells, stably expressing HCA587, were established as described in previous study and adopted for subsequent studies.

**FIGURE 4:**
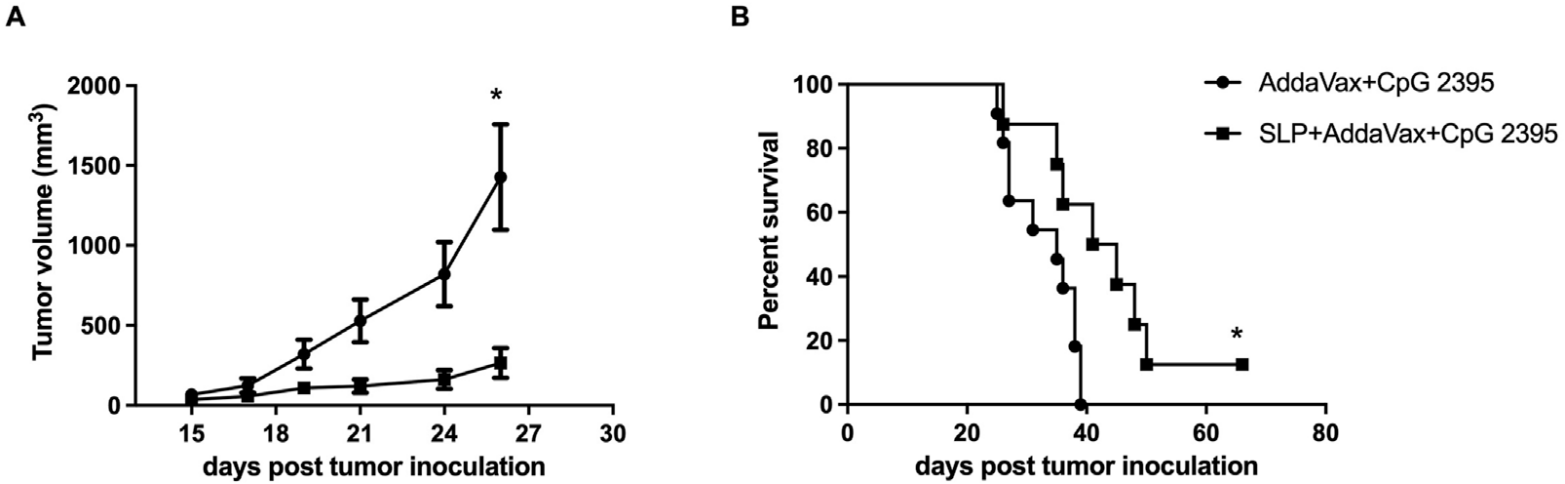
Therapeutic vaccination with HCA587 SLP assembled in AddaVax and CpG 2395 confers protection against HCA587-expressing tumors. 6-to-8-week female C57BL/6 mice were inoculated with 2 × 10^4^ B16-HCA587 cells, 1 day post tumor inoculation, one group of them were vaccinated with HCA587 SLP + AddaVax + CpG 2395; the other group received the treatment of AddaVax + CpG 2395 (adjuvant control). Mouse survival was monitored daily, and the tumor length and width were measured every 2–3 days with a caliper, and calculated using the following formula: (length × width^2^)/2. **(A)** Tumor size (n = 8–12 per group). Points represented mean of tumor volumes; bars represented SE. **(B)** Survival curve of tumor-bearing mice. *, *P* < 0.05.

C57BL/6 mice were inoculated with 2 × 10^4^ B16-HCA587 cells and immunized 1 day later with the HCA587 SLP in combination with AddaVax and CpG 2395 (HCA587 SLP + AddaVax + CpG 2395) or adjuvant control (AddaVax + CpG 2395). Compared with adjuvant control, HCA587 SLP vaccinated with AddaVax and CpG 2395 conferred efficient protection against tumor, as demonstrated by the retarded tumor growth (Figure 4A) and prolonged survival of the tumor-bearing mice (Figure 4B). These results indicated that the vaccination of HCA587 SLP in combination with AddaVax and CpG 2395 could provide sufficient anti-tumor effects in our studies.

## Discussion

HCA587, one kind of cancer/testis antigens, possesses strong immunogenicity and has been confirmed to exert efficient anti-tumor effects during previous research, making it an ideal candidate for specific cancer immunity. There are many advantages for synthetic long peptide in the cancer vaccine development. Besides the speed of manufacturing, purity and safety, SLPs contain epitopes that are recognized by helper (CD4^+^) and cytotoxic (CD8^+^) T cells and, in contrast to short peptides, may need to be processed and presented to T cells by professional antigen-presenting cells [25, 26].

In our previous study, synthetic long peptides (14 peptides of 25–35 amino acids long with an overlapping of 14 amino acids) representing the HCA587 protein from amino acids 136 to 373, were applied to the immunization experiment and therapeutic model in the mode of emulsion with FA and CpG 1826. Albeit the specific IFN-γ-producing CD4^+^ T cell response can be induced, but no sufficient protection was observed against developed HCA587-expressing tumors, which may be due to the deficiency of vaccine-elicited CD8^+^ T cell response. Pleiotropic functions of IFN-α, especially enhancement of immune responses, make it an optimal component in cancer vaccine preparation. We applied rmIFN-α in combination with the HCA587 SLP vaccine (HCA587 SLP + FA + CpG 1826) in C57BL/6 mice, thus the cellular immune responses were detected through IFNγ-secreting ELISPOT assay. To our surprise, the application of rmIFN-α did not improve the intensity of HCA57 SLP-specific immune response. Besides that, given the adverse side-effects caused by the vaccine formulation, the local administration of rmIFN-α may be inappropriate for further research.

AddaVax is a squalene-based oil-in-water nano-emulsion, proved to be more powerful in eliciting both cellular and humoral immune responses than water-in-oil emulsion such as Freud’s adjuvant, which is more adept at inducing humoral immunity. CpG 2395, belonging to CpG-C, displays its potent in inducing IFN-α production from pDC and activating B cells. The combined use of AddaVax and CpG 2395 could multiply their strengths in recruitment and activation of antigen presenting cells, thus enhancement of cellular immune responses. The encouraging research findings by Maynard et al. about HPV17 E6 synthetic long peptide vaccine formulated with CpG and AddaVax has brought us huge confidence in utilization of AddaVax and CpG 2395 for HCA587 SLP vaccination [20]. By applying the emulsion of HCA587 SLP and CpG 2395 with AddaVax, the SLP-specific IFN-γ-secreting CD4^+^ and CD8^+^ T cell responses were verified through ELISPOT and intracellular cytokine assay (ICCS). And during our ICCS experiment, we encountered an interesting phenomenon, the manifestation of IFN-γ secreted by CD8^+^ T cells depending on when to apply BFA, which is an inhibitor of protein trafficking from endoplasmic reticulum (ER) to Golgi apparatus often used in intracellular cytokine assay [27]. Untimely BFA addition may block the transport of SLP-derived peptide-MHCⅠ complex to the cell surface, which led to less primed IFN-γ-producing CD8^+^ T cells, just as scarce IFN-γ-secreting CD8^+^ T cells upon simultaneous incubation of BFA and SLP with splenocytes. The cross-priming of CD8^+^ T cells taking more procedures and longer time than activation of CD4^+^ T cells.

HCA587 SLP in combination with AddaVax and CpG 2395 provided sufficient protection against B16-HCA587 challenge in C57BL/6 mice, demonstrated by the decreased tumor growth and prolonged survival compared to the adjuvant group. After 55 days post tumor inoculation, there was only one tumor-bearing mouse alive, which received the HCA587 SLP combined with AddaVax and CpG 2395 treatment. To probe whether the SLP-specific immune response was the key to efficient anti-tumor effects, the splenocytes of this mouse were re-stimulated with SLP and the secretion of IFN-γ and Granzyme B were detected through ICCS, and finally robust production of Granzyme B in CD8^+^ T cells was monitored, whereas IFN-γ-secreting CD8^+^ T cells were rarely noticed (in supplementary data). Granzyme B is a serine-protease released by CD8^+^ T cells and natural killer cells during the cellular immune response and represents one of the two dominant mechanisms by which cytotoxic T cells mediate cancer cell death. Larimer et al. reported that Granzyme B PET Imaging could serve as a predictive biomarker of cancer immunotherapy Response [28].

In summary, the present study demonstrates that HCA587 SLP in combination with AddaVax and CpG 2395 can induce robust CD4^+^ T cell and CD8^+^ T cell responses and potent antitumor immunity in the mouse. This data supports a potential clinical application of this HCA587-SLP combination with AddaVax and CpG 2395 in the treatment of HCA587-expressing cancer patients.

## Data Availability

The original contributions presented in the study are included in the article/supplementary material, further inquiries can be directed to the corresponding author.
